# Combinations of Anti-Angiogenic Agents and Immune Checkpoint Inhibitors in Renal Cell Carcinoma: Best Option?

**DOI:** 10.3390/cancers15041048

**Published:** 2023-02-07

**Authors:** Estelle Granet-Vaissiere, Félix Lefort, Charlotte Domblides, Mathieu Larroquette, Alain Ravaud, Jean-Christophe Bernhard, Marine Gross-Goupil

**Affiliations:** 1 Department of Medical Oncology, University Hospital of Bordeaux, 33000 Bordeaux, France; 2 Faculty of Medicine, University of Bordeaux, 33000 Bordeaux, France; 3 ImmunoConcEpt, CNRS UMR 5164, Bordeaux University, 33076 Bordeaux, France; 4 Department of Urology, University Hospital of Bordeaux, 33000 Bordeaux, France

**Keywords:** renal cell carcinoma, tyrosine kinase inhibitor, immune checkpoint inhibitor, combination

## Abstract

**Simple Summary:**

To summarise the data on the combination of antiangiogenic and immune checkpoints in the treatment of clear-cell renal-cell carcinoma. In this review, we detail the physiopathological rationale of combining tyrosine kinase inhibitors and immunotherapy, and the in vitro and in vivo experiences that first suggested a synergistic effect between these two therapeutic targets. These pre-clinical data led to successful clinical trial that are reviewed in this article. Beyond the main outcomes of the pivotal trials, we describe the features of the different combinations (pembrolizumab-axitinib, pembrolizumab-lenvatinib and cabozantinib-nivolumab) that can help the clinicians to choose between them in routine practice. Eventually we discuss how this new paradigm of combinations will shape the future therapeutic strategies in the treatment of clear-cell renal-cell carcinoma.

**Abstract:**

Over the past decade, major advances have been made in the treatment of advanced and metastatic renal cell carcinomas, specifically clear cell carcinomas. For many years the optimal approach was sequential; thus, monotherapies [principally tyrosine kinase inhibitors (TKIs)] targeting angiogenesis until toxicity or progressive disease developed. The rationale was the common mechanisms of action of the targeting agents and avoidance of the risk of overlapping toxicities. Immune checkpoint inhibitors (ICIs) are effective monotherapies, and combinations thereof with anti-angiogenic agents were thus later considered. Synergistic interactions were reported in vitro. Clinical efficacy was evident in three pivotal phase III trials with axitinib-pembrolizumab, cabozantinib-nivolumab, and lenvatinib-pembrolizumab combinations. Two other combinations showed interesting results but did not improve overall survival. However, the data aided our understanding of the new therapeutic approaches. A combination of the ICIs nivolumab and ipilimumab was the first to evidence better progression-free and overall survival compared to sunitinib in patients with intermediate or unfavourable prognoses as evaluated by the International mRCC Database Consortium (IMDC). Here we focus on the TKI-ICI combinations, emphasising the rationale of their use and the clinical results. To date, no biomarker facilitating the selection of an optimal treatment by disease and patient status has been reported.

## 1. Introduction

Annually, renal cell carcinoma (RCC) affects over 430,000 patients and causes 179,000 deaths worldwide. RCC represents 3% of all adult cancers and is the twelfth most common solid cancer [1]. RCC incidence has increased over the past decades, in part attributable to the sensitivities afforded by computed tomography and magnetic resonance imaging. Kidney cancers are heterogeneous, thus of several histological subtypes, of which clear-cell RCC (ccRCC) is the most common [2]. Despite major progress in robotic surgery and partial nephrectomy, around one-third of patients with local disease at the time of diagnosis relapse. Also, about one-third of patients are diagnosed with advanced or metastatic disease; almost all require systemic treatment. Such treatments have improved greatly in the past 20 years. The first breakthrough occurred around 2005. The Von Hippel Lindau (VHL) mutation was associated with (first) a familial history of ccRCC and (later) sporadic disease. This encouraged the development of the first family of vascular endothelial growth factor (VEGF)-targeting agents such as bevacizumab; and tyrosine kinase inhibitors (TKIs), including sorafenib, sunitinib, pazopanib, (and later) axitinib, cabozantinib, and lenvatinib [3,4]. The second breakthrough, which occurred in the last decade, was the development of immune checkpoint inhibitors (ICIs). Ipilimumab is an anti-CTLA-4 antibody; nivolumab, avelumab, pembrolizumab, and atezolizumab are anti-PD-1/PD-L1 antibodies. Although the sequential approach remained the standard of care for almost 10 years when only kinase-targeting agents were available, combinations of either ICIs or an ICI and an anti-angiogenic agent later became standard [5]. The rationale was that synergies were evident in vitro, and clinical trials were successful. Here, we overview the preclinical and clinical data on synergy, provide information aiding treatment choice, and discuss how combination approaches will affect future therapeutic strategies.

## 2. Rationale of and Preclinical Data on Monotherapy Efficacies: Two Principal Pathways Are Implicated in RCC Development

### 2.1. Anti-Angiogenesis

Angiogenesis is a major hallmark of tumourigenesis, particularly in ccRCC; the tumour-suppressor *VHL* gene plays a central role; loss of function of at least one allele is apparent in up to 90% of sporadic ccRCC cases. [6,7] *VHL* inactivation usually develops 5–20 years before diagnosis [8]. The VHL protein is one of several substrates for a member of the E3 ubiquitin ligase family of proteins that degrades the α subunit of hypoxia-inducible factor (HIF). When VHL is inactivated, HIF-α accumulates and dimerises with the HIF-β subunit, increasing transcription of the genes encoding VEGF, platelet-derived growth factor (PDGF), and fibroblast growth factor (FGF). VEGF plays a leading role in the angiogenesis that contributes to tumour development and dissemination. This pathway remains a key target when treating ccRCC.

### 2.2. Immunogenicity

ccRCCs escape the immune system, as revealed by several clinical reports and in vitro studies. ccRCC is frequently associated with inflammation, which is negatively prognostic [9]. C-reactive protein is a biomarker of immune-system activity in tumour microenvironments; preoperative levels thereof and the kinetics before and after nephrectomy predicted the risk of metastatic relapse [10,11,12,13,14]. The many cases of spontaneous metastasis regression after the removal of the primary tumour also imply that the antitumour immune response plays a major role [15,16,17,18,19,20,21]. The underlying mechanisms are not well characterised, but they could involve tumour antigen release and reduction of the secretion of immunosuppressive factors by the primary tumour [22]. This has also been observed after stereotactic irradiation, the so-called “abscopal” effect, and the embolisation of primary tumours [23,24]. Immune system over-activation in response to cytoreductive nephrectomy is supported by reports of autoimmune disease rebounds in patients undergoing surgery [25] and tumour regression after systemic infection [26].

### 2.3. Rationale and Pre-Clinical Evidence for Synergy between TKIs and ICIs

Angiogenesis and immunity are complex processes; their many interactions increase the complexity further. Tumour vasculatures induce immunosuppression. In turn, immune cells affect angiogenesis during tumour progression [27]. In ccRCC, pro-angiogenic agents such as VEGF play major roles by activating VEGF-R proteins, including VEFG-R2, expressed predominantly in endothelial cells (ECs). VEGF modulates the innate and adaptive immune responses via at least four main mechanisms [28], including the immune downregulation of CD4+ and CD8+ T cells by reducing the numbers of early progenitors and inhibiting CD3+ T cell proliferation; inhibition of the maturation of dendritic cells and antigen-presenting cells; recruitment of immunomodulatory cells such as Tregs; and modulation of protein expression in ECs, and vascular permeability. After activation, ECs release matrix metalloproteinases (MMPs) that degrade the basement membrane, aiding tumour invasion [29]. ECs in tumour microenvironments (TMEs) express proteins that downregulate the immune system, including PDL-1, which binds to PD-1 on T cells [30]. Tumour cells produce VEGF-A, in turn increasing PD-1 and CTLA-4 levels on the surfaces of CD8+ cells, creating an immunosuppressive environment [31]. Angiogenesis aids the development of an immunosuppressive microenvironment in many ways, principally by inducing hypoxia. Abnormal/dysfunctional neovessels ensure that the TME is constantly hypoxic, triggering the release of cytokines and chemoattractants that contribute to the tumour infiltration of immunosuppressive immune cells. Hypoxia also increases the levels of CTLA-4 and TIM-3 on Tregs and PD-L1 on myeloid-derived stem cells.

Simultaneously, innate and adaptive immune cells affect neovascularisation. Several types of immunosuppressive cells in TMEs enhance angiogenesis. For example, myeloid-derived suppressor cells (MDSCs) release VEGF and thus promote angiogenesis by increasing the levels of IL- l0, MMP-9, and Bv8 [27]; and CD4+ Th2 T cells increase angiogenesis via interleukin secretion and recruitment of M2-like tumour-associated macrophages [32]. The TME also plays a crucial role in immunity. Tumour EC and immunosuppressive immune cells interact, inducing a vicious cycle that distorts the anti-tumour immune response and increases tumour development [33,34]. Inhibition of this negative crosstalk between immune suppression and angiogenesis may be of major therapeutic assistance, restoring normal vascularisation and reprogramming the immune system [27].

By 2005, it had been suggested that vessel normalisation induced by anti-angiogenic agents improved the effects of chemotherapy, immunotherapy, and radiotherapy [35,36,37]. It thus appeared that VEGF inhibitors and ICIs might act synergistically in TMEs [38]; an anti-VEGF TKI might restore normal vascularisation and tissue permeability, allowing the influx of immune cells into the tumour stroma; an ICI might restore the immune system of the TME.

Such drug combinations were tested in vivo in mice. In 2003, Nair et al. immunised mice against major proteins of angiogenesis (VEGF, VEGFR-2, and Tie2) and various tumour neoantigens (e.g., that encoded by the telomerase gene, thus TERT). In mice injected with melanoma and bladder cancer cell lines, the antitumour responses were stronger in doubly immunised mice than in those immunised with either tumour neoantigens or neoangiogenesis proteins. [39] Later, Yasuda et al. evaluated the effects of monoclonal antibody blockade of PD1 and VEGFR-2 in mice with colon cancer [40]. The synergistic actions of anti-VEGFR2 and anti-PD-1 downsized tumours to a greater extent than did the individual drugs, without excessive toxicity. The two-drug combination reduced neovascularisation, re-established normal vascularisation, allowed immune cells and anti-cancer drugs to attain the tumours, and facilitated infiltration of T cells into the TME, consistent with previous studies [41,42,43].

**Figure 1:**
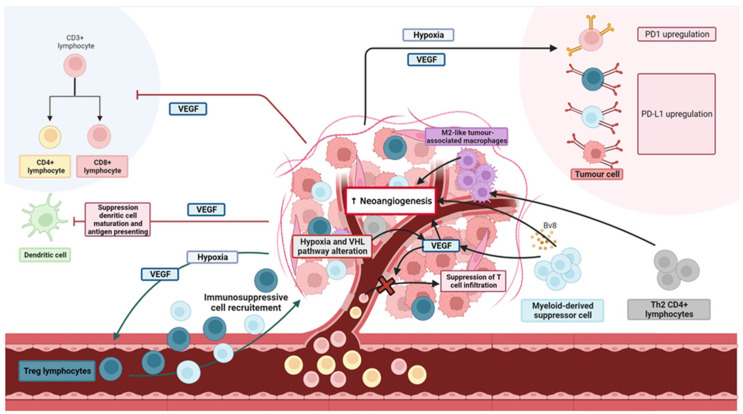
Main interactions between neoangiogenesis and immunosuppression. VEGF: vascular endothelial growth factor.

Thus, the data implied that antiangiogenic agents and ICIs might act synergistically. The main mechanisms implying this synergy, as described before, are summarised in Figure 1. Confirmatory clinical data followed; these are reviewed below.

## 3. Clinical Approach in First-Line Settings

### 3.1. The Past, Thus the Era of Monotherapy

For more than a decade, antiangiogenics such as sunitinib or pazopanib remained the standard of care for first-line treatment of metastatic ccRCC (mccRCC) [44,45]. Before 2007, treatments were based on the immunosuppressive agents interleukin-2 (IL-2) and interferon-alpha (INF-α) [46]; the latter inhibits tumour proliferation and stimulates mixed histocompatibility complex expression [47]. High doses of interleukin-2 induced complete responses in almost 10% of patients, but the toxicities were sometimes severe [48].

In 2007, sunitinib, a TKI targeting VEGF receptor types 1, 2, and 3; the PDGF-alpha receptor; c-KIT; and FLT3, was shown to be therapeutically useful. [49] The pivotal phase III trial included 750 patients. The median progression-free survival (PFS) was 11 months (95% confidence interval [CI] 11–13 months) in the sunitinib arm and 5 months in the control arm (95% CI 4–6 months; hazard ratio [HR] 0.53; 95% CI 0.451–0.643; *p* < 0.001). In terms of overall survival (OS), the superiority of sunitinib was also significant (HR 0.818; 95% CI 0.669–0.999; *p* < 0.049) [50]. The several adverse effects of sunitinib include increased blood pressure, asthenia, diarrhoea, and hand-foot syndrome. In 2010, pazopanib (another TKI targeting the VEGF 1, 2, and 3 receptors; the PDGF-α and β receptors; and c-Kit) was compared to a placebo in a phase III trial [51]. The median PFS was 9.2 months in the TKI arm versus 4.2 months in the placebo arm (HR 0.46; 95% CI 0.34–0.62; *p* < 0.0001) (39). In 2013, the COMPARZ phase III trial reported the non-inferiority of pazopanib compared to sunitinib in a first-line setting [44].

New TKIs were later developed. Axitinib, a second-generation TKI, selectively targets VEGF-R1, 2, and 3; it was not superior to sorafenib in terms of OS in a first-line setting but afforded a significantly longer PFS in a second-line setting [45]. Cabozantinib is a TKI with a broader spectrum of action, thus acting against the VEGF-Rs, the c-Met receptor, and AXL, which is implicated in the development of resistance to anti-angiogenic agents. After the demonstration of efficacy in the second-line setting of the METEOR trial [52], cabozantinib was compared to sunitinib in a first-line setting for patients with intermediate and poor prognoses in the phase II CABOSUN trial. The PFS in the cabozantinib arm was 8.2 months (95% CI 6.2–8.8 months) compared to 5.6 months in the sunitinib arm (95% CI 3.4–8.1 months; HR 0.66; *p* = 0.012). Cabozantinib has been approved by the US Food and Drug Administration (FDA) as a new treatment option in first-line settings. However, later analysis of trial data did not demonstrate any superiority of cabozantinib in terms of OS, which was 26.6 months in the cabozantinib arm and 21.2 months in the sunitinib arm (HR 0.80; 95% CI 0.53–1.21); the drug was not approved by the European Medicines Agency (EMA) in this indication [53].

The therapeutic benefits of ICIs were initially revealed in a second-line setting with nivolumab. In the Checkmate-025 phase III trial, nivolumab was compared to everolimus in 821 patients pre-treated with at least one anti-angiogenic agent [52]. Although no difference in PFS was observed, the ICI improved the overall response rate (ORR) (25% versus 5%, *p* < 0.001) and OS (HR 0.73; 95% CI 0.57–0.93; *p* = 0.002). This led to the approval of the first ICI for mRCC. Pembrolizumab was evaluated in the first-line Keynote-427 phase II trial that featured several cohorts, of which one contained 110 patients with RCC with good (37.3%), intermediate (47.3%), and poor (15.5%) prognoses as judged by the International mRCC Database Consortium (IMDC). The ORR was 36.4%, and the PFS was 7.1 months [53].

### 3.2. The Present Era of Combination Therapy

The first combination approved in a first-line mccRCC setting was an ICI doublet, thus a nivolumab-ipilimumab combination. The Checkmate-214 trial demonstrated the superiority of the doublet compared to sunitinib in patients of intermediate and poor IMDC risk groups [54]. The doublet greatly enhanced OS. At the 5-year follow-up, the median OS was 55.7 months versus 38.4 months for sunitinib (HR 0.72 [0.62–0.85]) [55]. The ORR was also markedly increased (39%), as was the complete response (CR) (12%). However, progression was evident in 18% of patients; the combination did not increase PFS. The individual efficacies of TKIs and ICIs, their different modes of action, and their non-cumulative tolerance profiles implied that they should be combined as first-line treatments. Many studies have reported the efficacies of TKI-ICI combinations in mccRCC patients and their superiority compared to sunitinib in terms of both PFS and OS.

The adverse effects of anti-VEGF-R/TKI combinations can be severe and are principally arterial hypertension, diarrhoea, hand-foot syndrome, and hepatotoxicity. These are usually dose-dependent and may be managed via dose reduction. ICIs also evidence specific toxicities and must sometimes be interrupted or discontinued. Liver toxicity, diarrhoea, and dysthyroidism may be induced by both TKIs and ICIs, and require careful management [56]. All but two of the tested TKI-ICI combinations are similarly efficacious, but the tolerance profiles differ in terms of the incidences of adverse effects and severity.

Two trials failed to demonstrate the superiority of a combination over sunitinib; these were the Javelin renal 101 trial evaluating avelumab plus axitinib [57] and the IMmotion 151 trial evaluating bevacizumab plus atezolizumab [58]. In contrast, three pivotal phase III trials defined combinations as the new standards for first-line settings, thus the Keynote-426 (axitinib-pembrolizumab), the Checkmate-9ER (cabozantinib-nivolumab), and the CLEAR (lenvatinib-pembrolizumab) studies [59,60,61].

In 2019, the phase III KEYNOTE 426 study was the first to compare the efficacy of pembrolizumab-axitinib (200 mg/3 weeks; 5 mg twice a day; 7/7; adjusted in terms of tolerance) versus sunitinib (50 mg/day, 4/2 weeks) in treatment-naive mccRCC patients with favourable, intermediate, and unfavourable IMDC prognoses [59]. The combination was significantly superior in 861 randomised patients, both in terms of PFS (HR 0.69; 95% CI 0.57–0.84; *p* < 0.001) and OS (HR 0.53; 95% CI 0.38–0.74; *p* < 0.0001). The overall results were consistent across all study subgroups, including the subgroups differing in terms of IMDC prognoses and those varying in terms of PDL-1 expression levels.

Pembrolizumab-axitinib treatment was associated with side effects of grade 3 or higher in 75.8% of patients, triggering discontinuation of both drugs in 10.7% of patients. In the sunitinib group, 70.6% of patients experienced adverse events, leading to discontinuation in 49.9%. In both groups, the most frequent side effects were hypertension and diarrhoea. Four deaths from toxicity were reported in the pembrolizumab-axitinib arm and seven in the sunitinib arm. This pivotal phase III study defined the pembrolizumab-axitinib combination as the new standard of care for the first-line management of mccRCC, regardless of prognosis [62]. Only 2 years later, thus in 2021, the phase III CLEAR trial evaluated the efficacy of lenvatinib in combination with pembrolizumab (lenvatinib 20 mg/day; 7/7; pembrolizumab 200 mg/3 weeks) or everolimus (lenvatinib 18 mg/day; 7/7; everolimus 5 mg/day; 7/7), compared to standard sunitinib alone, in 1069 patients with first-line mccRCC with favourable, intermediate, or unfavourable prognoses according to the Memorial Sloan-Kettering Cancer Center (MSKCC) criteria [61]. An independent review committee found a significant improvement in the PFS (the primary objective). The median PFS was 23.9 months (95% CI 20.8–27.7 months) in the TKI-ICI combination arm versus 9.2 months (95% CI 6.0–11.0 months) in the sunitinib arm (HR 0.39; 95% CI 0.32–0.49; *p* < 0.001). In the other combination arm, the median PFS was 14.7 months (95% CI 11.1–16.7 months) (HR 0.65; 95% CI 0.53–0.80; *p* < 0.001). Such results were observed in all risk subgroups defined using the MSKCC and IMDC criteria. The OS data were immature, given the short follow-up. Nevertheless, the OS was significantly higher in the lenvatinib-pembrolizumab arm than in the sunitinib arm (HR 0.66; 95% CI 0.49–0.88; *p* = 0.005). Again, the combination was superior regardless of PDL1 status, except for patients at low IMDC risk. In terms of safety, the most common side-effect was diarrhoea in about two-thirds of the combination arm and half of the sunitinib arm. Grade 3 or higher side effects, including diarrhoea, hypertension, elevated blood lipid levels, and hypertriglyceridemia, occurred in 82.4% of the lenvatinib-pembrolizumab arm, 83.1% of the lenvatinib-everolimus arm, and 71.8% of the sunitinib arm, requiring discontinuation of lenvatinib and/or pembrolizumab in 78.4% and discontinuation of sunitinib in 53.8% of patients. These results aided approval of the combination and recognition of the new standard of care [62]. Finally, and simultaneously, a third pivotal phase III trial, Checkmate 9ER, compared the efficacy of a combination of nivolumab and cabozantinib (nivolumab 240 mg/2 weeks; cabozantinib 40 mg/day; 7/7) versus sunitinib in 651 treatment-naive mccRCC patients of all IMDC risk groups [60]. PFS (the primary endpoint) was significantly increased, with medians of 16.6 months (95% CI 12.5–24.9 months) in the combination arm and 8.3 months (95% CI 7.0–9.7 months) in the sunitinib arm (HR 0.51; 95% CI 0.41–0.64; *p* < 0.001). OS was the secondary endpoint; the nivolumab-cabozantinib combination was also superior, with a 12-month OS of 85.7% (95% CI 81.3–89.1%) compared to 75.6% for sunitinib (95% CI 70.5–80.0%; HR 0.60; 95% CI 0.40–0.89; *p* = 0.001), as was also the case for the ORR (55.7%; 95% CI 50.1–61.2% versus 27.1%; 95% CI 22.4–32.3%; *p* < 0.001). The benefit of the combination was consistent across all subgroups, including the IMDC subgroups and those varying in terms of PDL-1 expression. In the safety context, 60.6% of patients in the nivolumab-cabozantinib arm and 50.9% in the sunitinib arm experienced grade 3 or higher treatment-related side effects; hypertension; hand-foot syndrome; asthenia; and liver, pancreatic, haematological, and ionic disturbances were the most common in both arms.

Other TKI-ICI combinations have also been evaluated. Those featuring sunitinib or pazopanib with nivolumab in the CHECKMATE-016 trial were associated with non-acceptable toxicities, thus grade 3 in 80% of patients and grade 4 in 70%, particularly liver toxicities [63]. Such toxicities were also evident in a phase I/II study evaluating the safety and efficacy of pazopanib in combination with pembrolizumab (90% grade 3 or 4 toxicities, including liver injury) [64].

As stated above, atezolizumab-bevacizumab and avelumab-axitinib have been evaluated in the phase III IMmotion-150 and Javelin Renal-101 trials, respectively [58,65]. The first study failed to demonstrate any PFS superiority of the combination over sunitinib in a first-line setting of patients with ccRCC selected by reference to the PD-1 levels. The superiority of a combination of avelumab (10 mg/kg/2 weeks) and axitinib (5 mg twice daily; 7/7; dose-escalation permitted) in terms of PFS (compared to sunitinib) in the Javelin Renal 101 trial allowed the FDA to approve the combination for previously untreated mccRCC patients regardless of MSKCC or IMDC status. The median PFS in patients with PD-L1-positive tumours (63.2%) was 13.8 months (95% CI 11.1–not estimated) in the combination arm versus 7.2 months (95% CI 5.7–9.7 months) in the control arm (HR 0.61; 95% CI 0.47–0.79; *p* < 0.001). However, on longer follow-up, the combination was not superior in terms of the OS of the 886 patients [66]. In terms of safety, side effects of all grades occurred in more than 99% of cases in both treatment groups, including 38.2% of all cases receiving immunotherapy. Side effects of grade 3 or higher developed in 71.2% of the avelumab-axitinib group, leading to treatment discontinuation in 7.6% and in 71.5% of the sunitinib group, leading to discontinuation in 13.4%. The most frequent side effects in the avelumab-axitinib group were diarrhoea and hypertension.

The Immotion-151 trial focused on a bevacizumab and atezolizumab combination in a first-line setting of patients with PD-L1-positive tumours. Although the combination evidenced a favourable toxicity profile, notably for bevacizumab, the trial failed to show the superiority of the combination compared to sunitinib in terms of either PFS or OS.

The key characteristics and results of positive phase III trials are summarised in Table 1 and Table 2. To date, pembrolizumab-axitinib, pembrolizumab-lenvatinib, and cabozantinib-nivolumab have not been compared in a prospective randomised trial. The therapeutic objectives of such trials should be the CR rate and PFS.

## 4. Synergistic Toxicity

Although combination therapies are effective against mccRCC, toxicities have increased. The mechanisms of ICI- and TKI-associated adverse events are very different. ICI toxicities are caused by nonspecific activation of the immune system, whereas TKI adverse events are caused by distinct mechanisms [56]. If adverse events are largely imputable to one or the other drug, ICIs and TKIs sometimes interact to increase side effects. The most striking example is liver toxicity. In the Keynote 426 trial, the incidences of grade 3 and 4 liver enzyme elevations (20 and 13%, respectively) were higher than observed during pembrolizumab monotherapy for other tumours (1.8–4.8% grade 3 and 1.6–4.1% grade 4 toxicities) or axitinib monotherapy for mccRCC (2% grade 3 and 2% grade 4) [68]. Thus, the liver toxicities seem to be not additive but rather synergistic, as emphasised by the toxicities of combinations using pazopanib, a TKI associated with a high incidence of liver toxicity. In the COMPARZ trial, grade 3 or 4 increases in alanine aminotransferase (ALT) levels were reported in 17.6% of patients who received pazopanib versus 3.9% of those who received sunitinib [44]. In a phase I/II study evaluating pazopanib in combination with pembrolizumab, grade 3–4 ALT elevations were reported in 50% and 60% of the patients on pembrolizumab plus pazopanib 600 mg or pazopanib 800 mg, respectively [64]. To the best of our knowledge, the mechanisms involved remain unknown. Physicians must choose combinations wisely and be vigilant in terms of side effects, especially in real-world patients.

## 5. Selection Criteria: Therapeutic Objectives and Clinical Outcomes

There are two indisputable combination selection criteria in first-line settings. The FDA and EMA have approved pembrolizumab-axitinib, cabozantinib-nivolumab, and lenvatinib-pembrolizumab for all IMDC subgroups, but nivolumab and ipilimumab only for those with intermediate and poor IMDC prognoses. Physicians must be guided by this. Second, the tolerance profiles and counter-indications to certain drug combinations must be carefully reviewed. Safety data that aid decision-making are summarised in Table 3. Other selection criteria are more controversial because they are based on indirect comparisons of phase III randomised trials or on conflicting and/or biased retrospective data. However, in practice, the choice of treatment is also guided by the objectives of physicians.

## 6. What Is More Important? Any Response, a Long Response, or a Favourable Safety Profile? Some Suggestions Follow

- For patients with a rapidly progressive life-threatening disease, it is essential to avoid upfront progression. A TKI-ICI combination is preferred given the lower rate of refractory disease observed in those on lenvatinib plus pembrolizumab or cabozantinib plus nivolumab (at best 5% and 6% of those with progressive disease, respectively) [60,61];
- In contrast, durable responses are afforded by nivolumab plus ipilimumab (PFS 36% at 2 years and 31% at 4 years) [69]. Although this “plateau effect” may reflect the longer follow-up of the Checkmate 214 trial than other trials, an ICI–ICI combination seems to be a good option for patients of intermediate/poor IMDC status with no life-threatening lesion;
- CR may reflect the curative potential of treatment. The CR rate is around 10% for the vast majority of the combinations [54,59,60], with an interesting 16% for the lenvatinib-pembrolizumab combination [61] and a disappointed 3% for the triplet cabozantinib-nivolumab-ipilimumab [68];
- For patients with brain metastases, cabozantinib afforded promising results, even in the absence of brain-directed local therapy [70]. The phase II CABRAMET Trial (NCT03967522) is currently recruiting patients with metastases to evaluate the intra- and extra-cranial effects of cabozantinib in a second-line real-world scenario;
- For patients with bone metastases, cabozantinib facilitated bone remodelling in preclinical studies, even when used as monotherapy [71], and was consistently better than sunitinib in patients with bone metastases in the Checkmate 9ER trial;
- For patients with sarcomatoid components, nivolumab plus ipilimumab may afford a good complete response and prolong survival [72,73].

## 7. The Future: New Strategies

Other drug combinations are in ongoing trials. Two explore the utilities of ICIs after initial progression. For example, the CONTACT-03 phase III clinical trial evaluates cabozantinib with and without atezolizumab in patients previously exposed to ICI therapy (NCT04338269). This study is enrolling not only ccRCC patients but also those with papillary or unclassified carcinomas. Even in second- or third-line settings, these data will improve our knowledge of the tolerabilities and efficacies of TKI-ICI combinations. Also, tivozanib, a selective and potent TKI targeting VEGF-R 1-3, is being tested in drug combinations. This TKI failed to demonstrate superiority to sorafenib in a first-line setting, despite a very favourable toxicity profile [74]. Later, the combination of tivozanib and nivolumab was evaluated in a small phase Ib/II trial in a first-line setting for half of the 25 patients and in a second-line setting for most of the others [75]. The overall response rate was 56%, and the disease-control rate was 96%. The median PFS was 18.9 months after a median follow-up of 19 months [75]. The toxicity profile appears reasonable, although 80% of patients developed grade 3/4 adverse events, particularly hypertension. The tivozanib-nivolumab combination is being evaluated (compared to tivozanib monotherapy) in the phase III TINIVO-02 trial and will yield useful data on drug efficacy and tolerability in patients previously exposed to ICIs (NCT04987203).

Therapeutic escalation is being appropriately tested. The COSMIC-313 trial is reinforcing immunotherapy with a double ICI combination (an ipilimumab-nivolumab backbone) and evaluating the benefit of adding cabozantinib (compared to placebo). Data were presented at the recent ESMO congress [67]. The primary objective data were positive; these were the PFS values of 855 first-line patients at intermediate and high risks. The median PFS was not attained in the triplet arm (14 months; NE) and was 11.3 months (7.7–18.2 months) for sunitinib (HR 0.73; 95% CI 0.57–0.94; *p* = 0.013. Notably, significant PFS improvement was observed only in the intermediately prognostic IMDC subgroup (HR 0.63), not in the poorly prognostic subgroup (HR 1.04). One explanation may be that the severe toxicity of the triplet triggers discontinuation or dose reductions. Thus, only 58% of the patients randomised to the triplet arm received the four planned doses of ipilimumab, compared to 73% of the nivolumab-ipilimumab-placebo arm. Of all patients, 90% and 70%, respectively, required dose reductions; cabozantinib and placebo reductions were required by 54% and 20%, respectively. Liver toxicity was the most frequent and severe adverse effect; 26% and 20% of patients evidenced grade 3–4 elevations in ALT and aspartate aminotransferase, respectively. The OS data are immature.

Two other trials are considering escalation. The first is also an upfront triplet combination examining the efficacy and safety of pembrolizumab plus belzutifan (an anti-HIF2α agent) plus lenvatinib or pembrolizumab/quavonlimab, an anti-CTLA4 ICI plus lenvatinib versus pembrolizumab plus lenvatinib as first-line treatments (NCT04736706). The second study uses a risk-adapted strategy, commencing with a nivolumab-ipilimumab combination with a plan to continue nivolumab in responders but to switch to cabozantinib in those with progressive disease, finally randomising all patients, thus responding or stabilised to nivolumab-cabozantinib or nivolumab alone as the standard of care (NCT03793166).

In contrast, de-escalation strategies are being evaluated in two French, multicentric, prospective, randomised phase III trials. The MOIO trial focuses on the possibility of decreasing the dose of ICI via increasing the interval of administration after controlling disease at 6 months using a classical regimen, thus an ICI–ICI or an ICI–TKI combination (NCT05078047). Another form of de-escalation is anticipated interruption of treatment. This approach is being evaluated in the SPICI trial (NCT05219318). After 1 year of ICI–TKI combination treatment, 372 patients at good or intermediate risk (thus with only one risk factor) who have responded will be randomised to discontinuation or continued treatment for 2 years. Mains phase III ongoing trials concerning ICI-TKI combinations strategies are summarised in Table 4.

## 8. Discussion

We have shown that the effects of ICIs and TKIs are not cumulative but rather synergistic. There is a plausible physiopathological rationale. The data have been validated in the laboratory and the clinic. After a decade of sequential monotherapies, principally anti-angiogenic TKIs, combination therapies are now the standard of care for all first-line mccRCC patients. ICI–TKI combinations are appropriate for all patients, but ICI–ICI combinations are appropriate only for those with intermediate and poor prognoses [62]. This paradigm shift raises a question: What lies ahead in terms of mccRCC management? Currently, the burning question is: how can the best drug combination be identified? The Checkmate-214 trial emphasised the efficacy of anti-angiogenic TKIs such as sunitinib (even as monotherapies) in patients with good prognoses [54]. In other subgroups, the choice of an ICI–TKI combination can be guided by various criteria, such as the lowest progression rate, the PFS, or the remission rate (including CR) in patients with aggressive and symptomatic disease. However, the appropriate goal may be a long OS or a long duration of treatment-free survival. Then, an ICI–ICI combination may be best for patients with the non-explosive disease. The toxicity profiles, especially those of ICI–TKI combinations, may also be relevant.

Unfortunately, no predictive biomarker aiding the selection of an optimal drug combination is yet available. The role played by PD-L1 is less clear in ccRCC than in other cancers. Indeed, a clinical benefit in terms of OS was reported even in patients with low-level PD-L1 expression receiving the ICI–ICI combination in the Checkmate-214 trial [76] and the ICI–TKI combination of the Keynote-426 trial [59]. Nevertheless, as reported by Mori et al., tumour expression of PD-L1 is associated with an increased ORR and a prolonged PFS in mccRCC patients receiving ICIs [77]. Unfortunately, this does not mean that an ICI–ICI combination is necessarily better than an ICI–TKI combination. Also, the studies vary greatly in terms of the markers measured, the assays used, and the evaluations of tumour and/or immune cells [78].

The molecular classification developed in the IMmotion 150 trial and validated in the IMmotion 151 trial defines tumour subgroups as highly or less angiogenic and/or immunogenic [79,80,81]. This is not routinely possible in practice. BIONIKK is the only prospective trial conducted to date; treatment was chosen on the basis of the tumour molecular phenotype [82]. Such interesting results, unfortunately, cannot be applied in clinical practice. Furthermore, a tool allowing selection from among ICI–ICI combinations, or sunitinib or ICI (nivolumab) monotherapies, has been devised. In contrast to other tumours, the RCC tumour mutational burden does not predict the efficacy of ICIs [83].

The efficacies of combination therapies in first-line mccRCC settings imply that useful drug combinations not only for such settings but also for later in the course of the disease remain to be discovered. Many ongoing trials of new drugs seek potential synergies. For example, one phase II study enrolling mccRCC patients after prior ICI therapy is evaluating axitinib plus PFOX (an OX40 agonist antibody) or placebo and has revealed a trend (albeit non-significant) toward a better PFS with the combination treatment (median PFS 13.2 vs. 8.5 months; HR 0.85; 95% CI 0.45–1.60) [84]. The phase III MK6482-012 trial compares belzutifan (an HIF2-α inhibitor) plus pembrolizumab plus lenvatinib or pembrolizumab plus quavonlimab (an anti-CTLA4 agent) plus lenvatinib to pembrolizumab plus lenvatinib in a first-line setting of mccRCC patients (NCT04736706).

## 9. Conclusions

After many years of sequential monotherapies, combination strategies now prolong the survival of mccRCC patients and have become the standard of care in first-line settings. This new paradigm raises many issues in terms of patient selection criteria, cost-effectiveness, toxicity management, further options, and dose intensification or de-escalation. The next challenge is personalised mccRCC medicine.

## Figures and Tables

**Table 1:** The principal design features and patient characteristics of phase III trials evaluating dual ICI and ICI-TKI combinations in first-line mccRCC settings.

Study	Treatment Arms	Patients (No.)	Primary Outcomes	IMDC Group (%)	Previous Nephrectomy (%)	Sarcomatoid Features (%)	Bone Metastasis Status	Liver Metastasis Status	PD-L1 Expression ≥ 1% (Score)
CheckMate 214 [54]	IPI + NIVO vs. SUN	425 vs. 422	PFS OS and ORR (intermediate/poor risk patients) (IRC)	Favourable: 23 Intermediate: 61 Poor: 16	82	17	20	18	23 *
KEYNOTE-426 [59]	AXI + PEMBRO vs. SUN	432 vs. 429	PFS (BICR) and OS in an ITT population	Favourable: 32 Intermediate: 55 Poor: 13	83	18	24	15	59 **
CheckMate 9ER [60]	CABO + NIVO vs. SUN	323 vs. 328	PFS (BICR) in an ITT population	Favourable: 23 Intermediate: 58 Poor: 19	69	11	24	23	26 *
CLEAR [61]	PEMBRO + LENVA vs. SUN	335 vs. 357	PFS (IRC) in an ITT population	Favourable: 31 Intermediate: 52 Poor: 9	73	8	24	17	30 **
COSMIC-313 [67]	NIVO + IPI + CABO vs. NIVO + IPI	428 vs. 427	PFS (BICR)	Intermediate: 75 Poor: 25	65	NA	17	20	64 ***

Test used to determine PD-L1 status: *: Tumor Proportion Score (TPS). **: Combined Positive Score (CPS). ***: PD-L1 IHC 28-8 pharmDx test. Abbreviations: AXI: axitinib; BICR: blinded independent central review; CABO: cabozantinib; CPS: Combined Positive Score; IMDC: International Metastatic RCC Database Consortium; IPI: ipilimumab; IRC: independent radiology review committee; ITT: intention-to-treat; LENVA: Lenvatinib; NA: not available; NIVO: nivolumab; OS: overall survival; PEMBRO: pembrolizumab; PFS: progression-free survival; SUN: sunitinib.

**Table 2:** Available results of phase III trials evaluating dual ICI and ICI-TKI combinations in mccRCC first-line settings.

Study	Treatment Arms	Patients (No.)	IMDC Group	Follow-Up (Months); Median	PFS (Months); Median	OS (Months); Median	Complete Response (%)
CheckMate 214 [54]	IPI + NIVO vs. SUN	425 vs. 422	Intermediate and poor	67.7	11.2 vs. 8.3 HR 0.74 95% CI [0.62–0.88] *p* < 0.0004	48.1 vs. 26.6 HR 0.65 95% CI [0.54–0.78] *p* < 0.0001	10.4 vs. 1.4 *
KEYNOTE-426 [59]	AXI + PEMBRO vs. SUN	432 vs. 429	All	42.8	15.7 vs. 11.1 HR 0.68 95% CI [0.58–0.80] *p* < 0.0001	45.7 vs. 40.1 HR 0.73 95 CI [0.60–0.88] *p* < 0.001	10.0 vs. 3.5 **
CheckMate 9ER [60]	CABO + NIVO vs. SUN	323 vs. 328	All	23.5	16.6 vs. 8.3 HR 0.56 [95% CI 0.46–0.68] *p* < 0.0001	37.7 vs. 34.3 HR 0.70 95% CI [0.55–0.90] *p* = 0.004	12 vs. 5 **
CLEAR [61]	PEMBRO + LENVA vs. SUN	335 vs. 357	All	26.6	23.9 vs. 9.2 HR 0.39 [0.32–0.49] *p* < 0.001	NR vs. NR HR 0.66 [0.49–0.88] *p* = 0.005	16.1 vs. 4.2 *
COSMIC-313 [67]	NIVO + IPI + CABO vs. NIVO + IPI	428 vs. 427	All	20.2	NR vs. 11.3, HR 0.73 [0.57–0.94] *p* < 0.013	NR	3 vs. 3 **

*: independent radiology review committee **: blinded independent central review. Abbreviations: AXI: axitinib; CABO: cabozantinib; IMDC: International Metastatic RCC Database Consortium; IPI: ipilimumab; LENVA: lenvatinib; NIVO: nivolumab; OS: overall survival, PEMBRO: pembrolizumab; PFS: progression-free survival; HR: hazard ratio; 95% CI: 95% confidence interval; NR: not reported; SUN: sunitinib.

**Table 3:** The principal toxicities encountered in phase III trials evaluating dual ICI and ICI–TKI combinations in first-line mccRCC settings.

Study	Grade 3–4 AEs	Principal TRAEs (All Grades) in the Experimental Arms	Principal TRAEs (Grades 3–4) in the Experimental Arms	Dose Reduction	Drug Interruption	Drug Discontinuation
CheckMate 214 [54]	IPI + NIVO vs. SUN	Fatigue: 38% Pruritus: 29.3% Diarrhoea: 28.3% Rash: 22.7% Nausea: 20.1%	Lipase increase: 10.6% Hepatic: 8.6% Endocrine: 6.9% Gastrointestinal: 4.9% Skin: 3.8%	NA	IPI: 27.1% (85.3% because of AEs); NIVO: 58.3% (65.8% because of AEs)	21.6%
KEYNOTE-426 [59]	AXI + PEMBRO vs. SUN	Diarrhoea: 49% Hypertension: 41.7% Hypothyroidism: 31.5% Fatigue: 30.3% PPE: 27.7%	Hypertension: 21.2% ALT increase: 12.1% Diarrhoea: 7.2% PPE: 5.1% Proteinuria: 2.6%	AXI: 20% dose reduction because of drug-related AEs PEMBRO: NA	Any treatment: 69.9% PEMBRO and AXI 35.7% PEMBRO: 50.3% AXI: 63.9%	PEMBRO or AXI: 25.9% PEMBRO: 18.6% Both: 6.3%
CheckMate 9ER [60]	CABO + NIVO vs. SUN	Diarrhoea: 56.9% PPE: 38.1% Hypothyroidism: 33.4% Hypertension: 30.3% Fatigue: 26.9%	Hypertension: 10.9% PPE: 7.5% Hyponatremia: 6.9% Diarrhoea: 5.6% Lipase increase: 5.3% Hypophosphoremia: 5.3%	CABO: 59.4%	Any treatment: 89.4% NIVO: 73.1% CABO: 81.9%	CABO or NIVO: 23.4% CABO: 7.2% NIVO: 9.7% Both: 5.0%
CLEAR [61]	PEMBRO + LENVA vs. SUN	Diarrhoea: 61.4% Hypertension: 55.4% Hypothyroidism: 47.2% Decreased appetite: 40.3% Fatigue: 40.1%	Hypertension: 27.6% Lipase increase: 12.8% Diarrhoea: 9.7% Weight decrease: 8% Proteinuria: 7.7%	LENVA: 68.8%	LENVA or PEMBRO: 78.4% LENVA: 73.0% PEMBRO: 55.1% Both: 39.2%	LENVA or PEMBRO: 37.2% LENVA: 25.6% PEMBRO: 28.7% 13.4%
COSMIC-313 [67]	NIVO + IPI + CABO vs. NIVO + IPI	ALT elevation: 46% AST elevation: 44% Diarrhoea: 41% PPE: 28% Hypothyroidism: 24% Hypertension: 23%	ALT elevation: 26% AST elevation: 20% Lipase increase: 9% Hypertension: 8%	CABO: 54%	Any treatment: 90%	Any treatment: 45% CABO or placebo: 28% NIVO: 26% IPI: 30% All: 12%

AXI: axitinib; CABO: cabozantinib; IPI: ipilimumab; LENVA: lenvatinib; NA: not applicable; NIVO: nivolumab; PEMBRO: pembrolizumab; PPE: palmar-plantar erythrodysesthesia; SUN: sunitinib; TRAEs: treatment-related adverse events.

**Table 4:** Ongoing phase III trials evaluating dual ICI and ICI-TKI combinations in mccRCC.

Study Name	Main Characteristics	Population	Experimental Arm	Comparator Arm	Primary Endpoint	Recruitment Status	Study Number
Escalation strategy							
MK6482-012	First line	1431	Belzutifan + pembrolizumab + lenvatinib and pembrolizumab + quavonlimab + lenvatinib	Pembrolizumab + lenvatinib	PFS, OS	Recruiting	NCT04736706
PDIGREE	First line (int/poor IMDC) According to the response after 4 cycles of nivolumab + ipilimumab	1046	Non-CR/NonPD cabozantinib + nivolumab CR: Nivolumab PD: Cabozantinib	NonCR/Non-PD CR: Nivolumab PD: Cabozantinib	OS	Recruiting	NCT03793166
COSMIC-313	First line (int/poor IMDC)	855	Nivolumab + Ipilimumab + Cabozantinib	Nivolumab + Ipilimumab	PFS	Active, not recruiting	NCT03937219
De-escalation strategy							
MOIO	First line	646	Standard dose intensity ICI	Reduced dose intensity ICI every 3 months	PFS	Recruiting	NCT05078047
SPICI	First line (fav/int with one IMDC fav criteria only) With OR at 12 Months with PD1/ICI + TKI	372	Treatment Pause	Treatment continuation	PFR	Not yet recruiting	NCT05219318
Rechallenge							
CONTACT-03	Post-anti PD(L)1	523	Cabozantinib + atezolizumab	Cabozantinib	PFS, OS	Active, not recruiting	NCT04338269
TiNivo-2	Second/Third line after ICI	326	Tivozanib + Nivolumab	Tivozanib	PFS	Recruiting	NCT04987203

CR: complete response; Fav: favourable; Int: intermediate; ICI: immune checkpoint inhibitor; IMDC: International Metastatic RCC Database Consortium; OS: overall survival; PFS: progression-free survival; PFR: progression-free rate; PD: progressive disease; TKI: tyrosine kinase inhibitor.
